# Effect of severity and etiology of chronic kidney disease in patients with heart failure with mildly reduced ejection fraction

**DOI:** 10.1007/s00392-024-02453-y

**Published:** 2024-05-06

**Authors:** Tobias Schupp, Kathrin Weidner, Felix Lau, Jan Forner, Alexander Schmitt, Marielen Reinhardt, Noah Abel, Niklas Ayasse, Thomas Bertsch, Muharrem Akin, Christel Weiß, Ibrahim Akin, Michael Behnes

**Affiliations:** 1 Department of Cardiology, Angiology, Haemostaseology and Medical Intensive Care, University Medical Centre Mannheim, Medical Faculty Mannheim, Heidelberg University, Mannheim, Germany; 2 Vth Department of Medicine (Nephrology, Hypertensiology, Endocrinology, Rheumatology, Pneumology) & Transplant Center Mannheim, University Hospital Mannheim, Medical Faculty Mannheim, University of Heidelberg, Heidelberg, Germany; 3 Institute of Clinical Chemistry, Laboratory Medicine and Transfusion Medicine, Nuremberg General Hospital, Paracelsus Medical University, Nuremberg, Germany; 4 Department of Cardiology, St. Josef-Hospital, Ruhr-Universität Bochum, 44791 Bochum, Germany; 5 Department of Statistical Analysis, Faculty of Medicine Mannheim, University of Heidelberg, Mannheim, Germany

**Keywords:** Heart failure with mildly reduced ejection fraction, HFmrEF, Chronic kidney disease, CKD, Mortality

## Abstract

**Objective:**

The study investigates the prognostic impact of the severity and etiology of chronic kidney disease (CKD) in patients with heart failure with mildly reduced ejection fraction (HFmrEF).

**Background:**

Data regarding the outcomes in patients with CKD in HFmrEF is scarce.

**Methods:**

Consecutive patients with HFmrEF were retrospectively included at one institution from 2016 to 2022. Prognosis of patients with different stages and etiologies of CKD was investigated with regard to the primary endpoint of all-cause mortality at 30 months.

**Results:**

A total of 2155 consecutive patients with HFmrEF were included with an overall prevalence of CKD of 31%. Even milder stages of CKD (i.e., KDIGO stage 3a) were associated with an increased risk of 30-months all-cause mortality (HR = 1.242; 95% CI 1.147–1.346; *p* = 0.001). However, long-term prognosis did not differ in patients with KDIGO stage 5 compared to patients with stage 4 (HR = 0.886; 95% CI 0.616–1.275; *p* = 0.515). Furthermore, the highest risk of HF-related rehospitalization was observed in patients with KDIGO stages 3b and 4 (log rank *p* ≤ 0.015), whereas patients with KDIGO stage 5 had a lower risk of HF-related rehospitalization compared to patients with KDIGO stage 4 (HR = 0.440; 95% CI 0.228–0.849; *p* = 0.014). In contrast, the etiology of CKD was not associated with the risk of 30-month all-cause mortality (log rank *p* ≥ 0.347) and HF-related rehospitalization (log rank *p* ≥ 0.149).

**Conclusion:**

In patients with HFmrEF, even milder stages of CKD were independently associated with increased risk of 30-months all-cause mortality.

**Supplementary Information:**

The online version contains supplementary material available at 10.1007/s00392-024-02453-y.

## Introduction

Related to improved revascularization techniques and materials, as well as increasing supply with invasive cardiac devices (such as implantable cardioverter defibrillators (ICD) or resynchronization therapies) and cardiac pharmacotherapies, overall survival rates in patients with heart failure (HF) are steadily improving [1–4]. As a result of improved treatment strategies for patients with HF, characteristics of patients with HF have consistently changed during the past decades, leading into a higher proportion of older patients with increased burden with cardiac and non-cardiac comorbidities [5, 6]. Chronic kidney disease (CKD) was demonstrated to be an independent risk factor of HF progression and sudden cardiac death (SCD), whereas more than half of patients with CKD die due to cardiovascular diseases [7–9]. However, the prevalence and prognostic impact of CKD may vary among different HF entities. For instance, the presence of CKD revealed higher prognostic accuracy in patients with HF with reduced (HFrEF) than in HF with preserved left ventricular ejection fraction (LVEF) (HFpEF) within the “Swedish Heart Failure Registry.”

Following the introduction of HF with mildly reduced ejection fraction (HFmrEF) — which are characterized by a LVEF 41–49% as an independent category of HF patients, a scientific gap was created with limited data concerning the effect of comorbidities and treatment strategies for these HF patients [10, 11]. HFmrEF was shown to share features with both HFrEF (i.e., higher rate of males, increased prevalence of CAD) and HFpEF (i.e., higher cardiovascular risk profiles) [12–14]. However, data concerning the prevalence and prognostic impact of CKD in patients with HFmrEF, specifically with regard to the stage and etiology of CKD, remains limited [12, 15].

Therefore, the present study aims to [1] investigate the prognostic impact of different stages of CKD [2], the impact of different CKD etiologies, and [3] the effect of CKD stratified by important subgroups of patients with HFmrEF, using a large dataset of consecutive patients hospitalized with HFmrEF from 2016 to 2022.

## Methods

### Study patients, design, and data collection

For the present study, all consecutive patients hospitalized with HFmrEF at one university medical centre were included from January 2016 to December 2022 [16]. Using the electronic hospital information system, all relevant clinical data related to the index event were documented, such as baseline characteristics, vital signs on admission, prior medical history, prior medical treatment, length of index hospital and intensive care unit (ICU) stay, laboratory values, data derived from all non-invasive or invasive cardiac diagnostics and device therapies, such as echocardiographic data, coronary angiography, data being derived from prior or newly implanted cardiac devices, and pharmacotherapies at discharge.

The present study is derived from the “Heart Failure With Mildly Reduced Ejection Fraction Registry” (HARMER), representing a retrospective single-center registry including consecutive patients with HFmrEF hospitalized at the University Medical Center Mannheim (UMM), Germany (clinicaltrials.gov identifier: NCT05603390). The registry was carried out according to the principles of the declaration of Helsinki and was approved by the medical ethics committee II of the Medical Faculty Mannheim, University of Heidelberg, Germany (ethical approval code: 2022–818).

### Inclusion and exclusion criteria

All consecutive patients with ≥ 18 years of age hospitalized with HFmrEF at one institution were included. The diagnosis of HFmrEF was determined according to the “2021 ESC Guidelines for the diagnosis and treatment of acute and chronic HF” [10]. Accordingly, all patients with LVEF 41–49% with symptoms and/or signs of HF were included. The presence of elevated amino-terminal prohormone of brain natriuretic peptide (NT-proBNP) levels and other evidence of structural heart disease were considered to make the diagnosis more likely but were not mandatory for diagnosis of HFmrEF. Transthoracic echocardiography was performed by cardiologists during routine clinical in accordance with current European guidelines [17, 18]. Patients without measurement of the estimated glomerular filtration rate (eGFR) were excluded for the present study.

### Risk stratification

For the present study, risk stratification was performed according to the presence and severity of CKD. CKD was defined as abnormalities of kidney function with implication for health accompanied with an estimated glomerular filtration rate (eGFR) < 60 ml/min/1.73 m^2^ (i.e., according to kidney disease improving global outcome KDIGO stages 3a–5) and a duration > 3 months. Therefore, a minimum of two eGFR measurements separated by at least 3 months was required for the diagnosis of CKD [19–21]. All available eGFR values prior to (ambulatory or hospitalized patients) and during index hospitalization were evaluated with regard to the definition of CKD. In patients admitted with acute decompensated HF, eGFR levels were considered following recompensation to decrease the risk to misclassify patients with acute kidney injury.

For the present study, risk stratification was performed according to different KDIGO stages, etiology of CKD, and in important pre-specified subgroups (i.e., ischemic vs. non-ischemic cardiomyopathy, as well as stratified by patients with stable (i.e., prior HFmrEF), deteriorated (i.e., prior HFpEF), and improved HF (i.e., prior HFrEF)).

### Measurement of creatinine and eGFR

During index hospitalization, creatinine determination was carried out predominantly from lithium heparinate plasma. This assay is a modification of the Jaffé method with blank correction and axis segment adjustment. This blank correction is used to minimize interferences with bilirubin. The assay was carried out on a clinical chemistry analyzer (Atellica CH 930, Siemens Healthineers, Erlangen, Germany). eGFR was estimated by using the CKD-EPI formula. This is more accurate compared to the MDRD formula in estimating the GFR in the threshold region of beginning renal insufficiency [22, 23].

### Study endpoints

The primary endpoint was all-cause mortality at 30 months (median follow-up). Secondary endpoints comprised in-hospital all-cause mortality, all-cause mortality at 12 months, rehospitalization for worsening HF, cardiac rehospitalization, acute myocardial infarction (AMI), stroke, coronary revascularization, major adverse cardiac and cerebrovascular events (MACCE), and changes in LVEF and NT-pro BNP levels during follow-up. All-cause mortality was documented using the electronic hospital information system and by directly contacting state resident registration offices (“bureau of mortality statistics”). Cardiac rehospitalization was defined as rehospitalization due to a primary cardiac condition, including worsening HF, AMI, coronary revascularization, and symptomatic atrial or ventricular arrhythmias. MACCE was defined as a composite of all-cause mortality, coronary revascularization, non-fatal AMI, and non-fatal stroke. All echocardiographic examinations of inpatients and outpatients treated at our institution were assessed and LVEF, and NT-pro BNP levels were documented for the intervals of 0–6 months, 6–12 months, 12–18 months, 18–24 months, and 24–30 months during routine follow-up to further investigate the prognostic role of CKD on LVEF and NT-pro BNP levels during follow-up.

### Statistical methods

Quantitative data is presented as mean ± standard error of the mean (SEM), median and interquartile range (IQR), and ranges depending on the distribution of the data. They were compared using the Student’s *t* test for normally distributed data or the Mann–Whitney *U* test for nonparametric data. Deviations from a Gaussian distribution were tested by the Kolmogorov–Smirnov test. Qualitative data is presented as absolute and relative frequencies and were compared using the chi-square test or the Fisher’s exact test, as appropriate. Kaplan–Meier analyses were performed stratified by the severity of CKD, as well as to investigate the etiology of CKD and the prognostic value of CKD in pre-specified subgroups. Univariable hazard ratios (HR) were given together with 95% confidence intervals. The prognostic impact of the severity of CKD was investigated within multivariable Cox regression models using the “forward selection” option.

The results of all statistical tests were considered significant at *p* ≤ 0.05. SPSS (Version 28, IBM, Armonk, NY) was used for statistics.

## Results

### Study population

A total of 2228 consecutive patients with HFmrEF were hospitalized at our institution from 2016 to 2022. Forty-four patients lost to follow-up, and 29 patients with no measurement of eGFR on admission were excluded (Supplemental Fig. 1; Flow chart). The final study cohort comprised 2155 patients with HFmrEF with a total prevalence of CKD of 31%. Among patients with CKD, most patients presented with KDIGO stage 3b (32.8%), followed by 3a (30.1%), whereas only 11.9% of patients had KDIGO stage 5. Renal replacement therapy was performed in all patients with CKD and KDIGO stage 5. Patients with CKD were older (median age 81 vs. 72 years; *p* = 0.001) and had higher rates of prior CAD (54.0% vs. 35.2%; *p* = 0.001), prior AMI (30.9% vs. 20.8%; *p* = 0.036), as well as higher prevalence of congestive HF (52.2% vs. 25.3%; *p* = 0.001) (Table 1). In line with this, patients with CKD had higher rates of cardiovascular risk factors including arterial hypertension (89.2% vs. 72.8%; *p* = 0.001), diabetes mellitus (47.5% vs. 31.5%; *p* = 0.001), and hyperlipidemia (34.7% vs. 28.3%; *p* = 0.001). Ischemic cardiomyopathy was the most common HF etiology in both groups (60.1% vs. 56.7%), followed by hypertensive cardiomyopathy (8.0% vs. 8.0%) and primary non-ischemic cardiomyopathies (7.3% vs. 6.7%; *p* = 0.062). Patients with CKD had lower hemoglobin levels (median 10.9 vs. 13.0 g/dl; *p* = 0.001) and lower platelet count (median 213 vs. 233 × 10^9^/l; *p* = 0.001). Finally, the prescription rates of beta-blockers (81.5% vs. 75.7%; *p* = 0.001), aldosterone antagonists (16.5% vs. 12.9%; *p* = 0.031), and loop diuretics (76.3% vs. 35.9%; *p* = 0.001) were higher in patients with CKD.

**Table 1:** Baseline characteristics

	Non-CKD (*n* = 1481)		CKD (*n* = 674)		*p* value
Age, median (IQR)	72	(61–81)	81	(74–86)	**0.001**
Male sex, *n* (%)	998	(67.4)	390	(57.9)	**0.001**
Body mass index, kg/m^2^, median (IQR)	26.7	(24.0–30.5)	26.2	(23.5–30.9)	0.409
Medical history, *n* (%)					
Coronary artery disease	521	(35.2)	364	(54.0)	**0.001**
Prior myocardial infarction	308	(20.8)	208	(30.9)	**0.036**
Prior PCI	352	(23.8)	249	(36.9)	**0.001**
Prior CABG	108	(7.3)	106	(15.7)	**0.001**
Prior valvular surgery	58	(3.9)	38	(5.6)	0.072
Congestive heart failure	375	(25.3)	352	(52.2)	**0.001**
Decompensated heart failure < 12 months	106	(7.2)	125	(18.5)	**0.001**
Peripheral artery disease	123	(8.3)	123	(18.2)	**0.001**
Stroke	212	(14.3)	118	(17.5)	0.056
Liver cirrhosis	19	(1.3)	28	(4.2)	**0.001**
Malignancy	215	(14.5)	115	(17.1)	0.128
COPD	148	(10.0)	111	(16.5)	**0.001**
Cardiovascular risk factors, *n* (%)					
Arterial hypertension	1078	(72.8)	601	(89.2)	**0.001**
Diabetes mellitus	466	(31.5)	320	(47.5)	**0.001**
Hyperlipidemia	419	(28.3)	234	(34.7)	**0.003**
Smoking					
Current	338	(22.8)	66	(9.8)	**0.001**
Former	236	(15.9)	148	(22.0)	**0.001**
Family history	157	(10.6)	42	(6.2)	**0.001**
Comorbidities at index hospitalization, *n* (%)					
Acute coronary syndrome					
Unstable angina	74	(5.0)	24	(3.6)	0.138
STEMI	161	(10.9)	14	(2.1)	**0.001**
NSTEMI	205	(13.8)	69	(10.2)	**0.020**
Acute decompensated heart failure	226	(15.3)	258	(38.3)	**0.001**
Cardiogenic shock	35	(2.4)	18	(2.7)	0.669
Atrial fibrillation	543	(36.7)	358	(53.1)	**0.001**
Cardiopulmonary resuscitation	39	(2.6)	14	(2.1)	0.440
Out-of-hospital	19	(1.3)	3	(0.4)	0.073
In-hospital	20	(1.4)	11	(1.6)	0.611
Stroke	232	(15.7)	66	(9.8)	**0.001**
Heart failure etiology, *n* (%)					
Ischemic cardiomyopathy	839	(56.7)	405	(60.1)	0.062
Non-ischemic cardiomyopathy	99	(6.7)	49	(7.3)	
Hypertensive cardiomyopathy	119	(8.0)	54	(8.0)	
Congenital heart disease	3	(0.2)	1	(0.1)	
Valvular heart disease	56	(3.8)	40	(5.9)	
Tachycardia associated	94	(6.3)	31	(4.6)	
Tachymyopathy	29	(2.0)	9	(1.3)	
Pacemaker-induced cardiomyopathy	12	(0.8)	7	(1.0)	
Unknown	259	(17.5)	87	(12.9)	
NYHA functional class, *n* (%)					
I/II	1177	(79.4)	383	(56.8)	**0.001**
III	215	(14.5)	191	(28.3)	
IV	89	(6.0)	100	(14.8)	
Baseline laboratory values, median (IQR)					
Creatinine, mg/dL	0.95 (0.79–1.11)		1.71 (1.37–2.51)		**0.001**
eGFR, mL/min/1.73 m^2^	77 (64–94)		37 (24–47)		**0.001**
Hemoglobin, g/dL	13.0 (11.2–14.4)		10.9 (9.5–12.6)		**0.001**
C-reactive protein, mg/L	11 (3–40)		19 (5–54)		**0.001**
NT-pro BNP, pg/mL	1915 (592–4034)		5394 (2471–12489)		**0.001**
Cardiac troponin I, µg/L	0.03 (0.02–0.26)		0.03 (0.02–0.13)		0.981
Medication at discharge, *n* (%)					
ACE-inhibitor	771	(53.5)	275	(43.1)	**0.001**
ARB	309	(21.4)	184	(28.8)	**0.001**
Beta-blocker	1092	(75.7)	520	(81.5)	**0.004**
Aldosterone antagonist	186	(12.9)	105	(16.5)	**0.031**
ARNI	13	(0.9)	10	(1.6)	0.181
SGLT2-inhibitor	65	(4.5)	16	(2.5)	**0.030**
Loop diuretics	518	(35.9)	487	(76.3)	**0.001**
Statin	1008	(69.9)	418	(65.5)	**0.047**
Digitalis	69	(4.8)	33	(5.2)	0.706
Amiodarone	24	(1.7)	32	(5.0)	**0.001**

*ACE*, angiotensin-converting-enzyme; *ARB*, angiotensin receptor blocker; *ARNI*, angiotensin receptor neprilysin inhibitor; *CABG*, coronary artery bypass grafting; *CKD*, chronic kidney disease; *COPD*, chronic obstructive pulmonary disease; *IQR*, interquartile range; *(N)STEMI*, non-ST-segment elevation myocardial infarction; *NT-pro BNP*, aminoterminal pro-B-type natriuretic peptide; *NYHA*, New York Heart Association; *PCI*, percutaneous coronary intervention; *SGLT2*, sodium-glucose-linked transporter 2. Level of significance *p* ≤ 0.05. Bold type indicates statistical significance

### Correlations of eGFR with clinical and laboratory data

During index hospitalization, eGFR correlated with hemoglobin (*r* = 0.340; *p* = 0.001), platelet count (*r* = 0.105; *p* = 0.001), total cholesterol (*r* = 0.147; *p* = 0.001), and LDL cholesterol (*r* = 0.195; *p* = 0.001), whereas an inverse correlation with age (*r* =  − 0.361; *p* = 0.001), potassium (*r* =  − 0.177; *p* = 0.001) and NT-proBNP (*r* =  − 0.141; *p* = 0.001) was observed (Supplemental Table 1).

### Prognostic impact of different stages of CKD in patients with HFmrEF

**Fig. 1:**
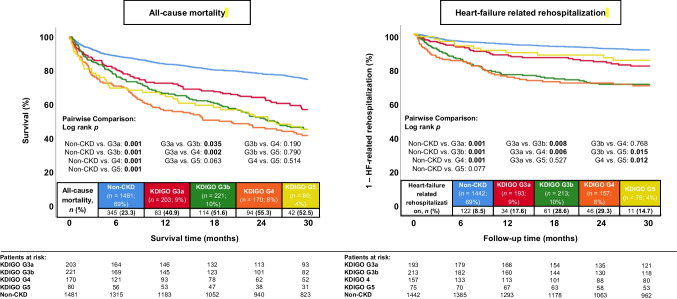
Kaplan–Meier analyses demonstrating the prognostic impact of different KDGIO stages on the primary endpoint all-cause mortality at 30 months (left panel), as well as on the risk of HF-related rehospitalization (right panel) within the entire study cohort

During a median follow-up of 30 months, the primary endpoint all-cause mortality occurred in 49.4% with CKD and in 23.3% without (Table 2: endpoints). Even milder stages of CKD (i.e., KDIGO stage 3a) were already associated with a higher risk of 30-month all-cause mortality compared to patients without CKD (HR = 1.242; 95% CI 1.147–1.346; *p* = 0.001) (Fig. 1; left panel). In line with this, patients with CKD and KDIGO stage 3b were associated with a higher risk of long-term all-cause mortality compared to patients with KDIGO stage 3a (HR = 1.833; 95% CI 1.041–3.229; *p* = 0.036). However, the long-term prognosis did not differ in patients with CKD and KDIGO stage 5 compared to patients with stage 4 (HR = 0.886; 95% CI 0.616–1.275; *p* = 0.515) or stage 3b (HR = 1.032; 95% CI 0.815–1.307; *p* = 0.791) Furthermore, the highest risk of HF-related rehospitalization was observed in patients with CKD and KDIGO stages 3b and 4 (log rank *p* ≤ 0.015), whereas patients with CKD and KDIGO stage 5 had a lower risk of HF-related rehospitalization compared to patients with KDIGO stage 4 (HR = 0.440; 95% CI 0.228–0.849; *p* = 0.014) and 3b (HR = 0.596; 95% CI 0.388–0.915; *p* = 0.018) (Fig. 1; right panel).

**Table 2:** Follow-up data, primary and secondary endpoints

	Non-CKD (*n* = 1481)		CKD (*n* = 674)		HR	95% CI	*p* value
Primary endpoint, *n* (%)							
All-cause mortality, at 30 months	345	(23.3)	333	(49.4)	2.498	2.149–2.905	**0.001**
Secondary endpoints, *n* (%)							
All-cause mortality, in-hospital	39	(2.6)	36	(5.3)	1.379	0.873–2.179	0.168
All-cause mortality, at 12 months	235	(15.9)	229	(34.0)	2.388	1.990–2.865	**0.001**
Heart-failure related rehospitalization, at 30 months	122	(8.5)	152	(23.8)	3.068	2.417–3.893	**0.001**
Cardiac rehospitalization, at 30 months	258	(17.9)	198	(31.0)	1.857	1.543–2.234	**0.001**
Revascularization, at 30 months	98	(6.8)	41	(6.4)	0.930	0.646–1.339	0.697
Acute myocardial infarction, at 30 months	34	(2.4)	28	(4.4)	1.840	1.116–3.034	**0.017**
Stroke, at 30 months	42	(2.9)	15	(2.4)	0.788	0.437–1.422	0.429
MACCE, at 30 months	466	(31.5)	367	(54.5)	2.002	1.746–2.296	**0.001**
Follow-up data, median (IQR)							
Hospitalization time, days	8 (5–14)		11 (7–19)		-	-	**0.001**
ICU time, days	0 (0–1)		0 (0–0)		-	-	**0.002**
Follow-up time, days	1024 (462–1805)		651 (198–1288)		-	-	**0.001**

*CI*, confidence interval; *CKD*, chronic kidney disease; *HR*, hazard ratio; *ICU*, intensive care unit; *IQR*, interquartile range; *MACCE*, major adverse cardiac and cerebrovascular events. Level of significance *p* ≤ 0.05. Bold type indicates statistical significance

After multivariable adjustment, already patients with KGIDO stage 3a had a higher risk of 30-month all-cause mortality compared to patients without CKD (HR = 1.331; 95% CI 1.016–1.744; *p* = 0.038) (Table 3). However, the risk of HF-related was specifically increased in patients with CKD and KDIGO stage 3b (HR = 2.058; 95% CI 1.444–2.933; *p* = 0.001) and 4 (HR = 1.912; 95% CI 1.274–2.869; *p* = 0.002), but not in patients with CKD and KDIGO stage 5 after multivariable adjustment (Table 3).

**Table 3:** Multivariable Cox regression analyses with regard to 30-month all-cause mortality and heart failure–related rehospitalization stratified by the severity of CKD

	All-cause mortality			Heart-failure related rehospitalization		
Variables	HR	95% CI	*p* value	HR	95% CI	*p* value
Age	1.038	1.029–1.046	**0.001**	1.006	0.993–1.019	0.372
Male sex	1.325	1.109–1.584	**0.002**	0.889	0.682–1.159	0.386
Body mass index	0.945	0.926–0.963	**0.001**	1.022	0.997–1.047	0.085
Prior congestive heart failure	1.114	0.906–1.369	0.305	1.506	1.104–2.055	**0.010**
Prior acute myocardial infarction	1.254	0.990–1.588	0.061	1.140	0.822–1.581	0.431
Decompensated heart failure < 12 months	0.869	0.657–1.149	0.323	1.308	0.926–1.847	0.128
Arterial hypertension	0.731	0.584–0.915	**0.006**	1.119	0.748–1.673	0.585
Diabetes mellitus	1.231	1.027–1.477	**0.025**	1.174	0.892–1.544	0.252
Ischemic cardiomyopathy	0.623	0.508–0.763	**0.001**	1.099	0.803–1.503	0.556
Right ventricular dysfunction	1.352	1.127–1.622	**0.001**	1.017	0.768–1.347	0.904
Atrial fibrillation	1.169	0.973–1.403	0.095	1.968	1.474–2.628	**0.001**
Acute decompensated heart failure	1.583	1.268–1.976	**0.001**	1.500	1.088–2.069	**0.013**
Cardiopulmonary resuscitation	1.722	1.006–2.947	**0.048**	1.110	0.353–3.491	0.859
NYHA functional class	1.019	0.922–1.128	0.707	1.244	1.071–1.445	**0.004**
Non-CKD	(Reference group)			(Reference group)		
KDIGO 3a	1.331	1.016–1.744	**0.038**	1.341	0.875–2.056	0.178
KDIGO 3b	1.611	1.256–2.066	**0.001**	2.058	1.444–2.933	**0.001**
KDIGO 4	1.786	1.349–2.364	**0.001**	1.912	1.274–2.869	**0.002**
KDIGO 5	2.608	1.740–3.909	**0.001**	1.446	0.725–2.884	0.295

*CI*, confidence interval; *CKD*, chronic kidney disease; *HR*, hazard ratio; *KDIGO*, kidney disease: improving global outcomes; *NYHA*, New York Heart Association. Level of significance *p* ≤ 0.05. Bold type indicates statistical significance

**Fig. 2:**
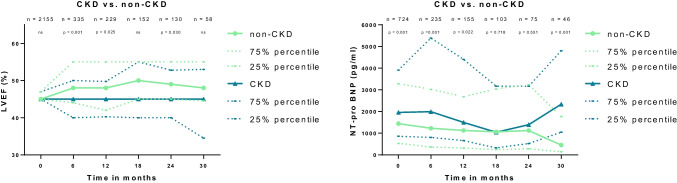
Changes in LVEF (left panel) and NT-pro BNP levels (right panel) among patients with and without CKD during 30 months of follow-up. Data is presented as median and interquartile range (IQR)

During follow-up, patients with CKD had a lower LVEF at 6 months (median 45 vs. 48; *p* = 0.001), at 12 months (median 45 vs. 48; *p* = 0.025), and at 24 months (median 45 vs. 49; *p* = 0.030) as compared to patients without CKD (Fig. 2; left panel). In line with this, NT-pro BNP levels were higher in patients with CKD during index hospitalization (median 1957 vs. 1441; *p* = 0.001), as well as at 6 months (median 1993 vs. 1226; *p* = 0.001), 12 months (median 1499 vs. 1126 *p* = 0.022) and 30 months (median 2337 vs. 452; *p* = 0.001) thereafter (Fig. 2; right panel).

### Prognostic impact of the etiology of CKD in HFmrEF

**Fig. 3:**
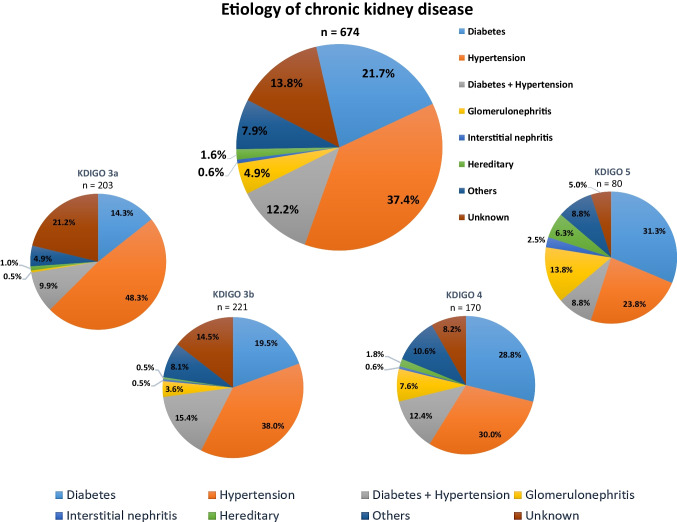
Distribution of the etiologies of CKD in patients with HFmrEF and concomitant CKD, as well as stratified by KDIGO stages

**Fig. 4:**
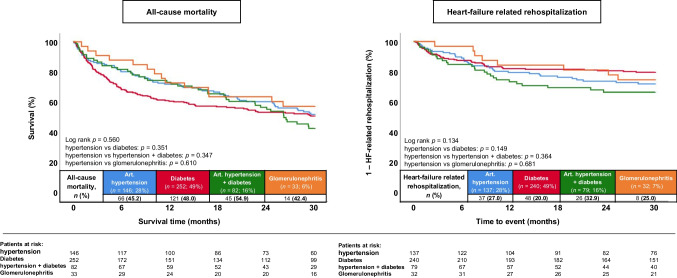
Kaplan–Meier analyses demonstrating the prognostic impact of different CKD etiologies on the primary endpoint all-cause mortality at 30 months (left panel), as well as on the risk of HF-related rehospitalization (right panel) in patients with CKD

In patients with HFmrEF, most patients had CKD related to arterial hypertension (38.4%), followed by diabetes mellitus (21.7%), whereas only 4.9% had CKD related to glomerulonephritis (Fig. 3). In patients with advanced stages of CKD (i.e., KDIGO stage 5), diabetes mellitus was the most common etiology of CKD in 31.3% of patients. However, the etiology of CKD was not associated with the risk of 30-month all-cause mortality (log rank *p* ≥ 0.347) and HF-related rehospitalization (log rank *p* ≥ 0.149) when comparing the prognosis of patients with the four most common etiologies of CKD (Fig. 4).

### Effect of CKD in pre-specified subgroups

Even when stratified by patients with ischemic and non-ischemic cardiomyopathy, patients with CKD and KDIGO stages 4 and 5 were associated with the highest risk of 30-month all-cause mortality, especially in patients with ischemic cardiomyopathy (stage 4 vs. 3b: log rank *p* = 0.010) (Supplemental Fig. 2). Interestingly, even patients with CKD and KDIGO stage 3a were associated with a higher risk of 30-month all-cause mortality compared to patients without CKD, which was observed in patients with ischemic (KDIGO stage 3a vs. non-CKD: HR = 1.273; 95% CI 1.138–1.424; *p* = 0.001) and non-ischemic cardiomyopathy (KDIGO stage 3a vs. non-CKD: HR = 1.211; 95% CI 1.080–1.358; *p* = 0.001). Furthermore, LVEF was lower in patients with CKD compared to patients without at 6 and 18 months of follow-up in patients with ischemic cardiomyopathy, whereas NT-proBNP levels were higher during index hospitalization and 6 months thereafter (Supplemental Fig. 3).

When stratified by prior LVEF, patients with CKD and KDIGO stage 3a were specifically associated with a higher risk of all-cause mortality in patients with stable HF (i.e., prior HFmrEF) (HR = 1.358; 95% CI 1.012–1.822; *p* = 0.041) and deteriorated HF (i.e., prior HFpEF) (HR = 1.164; 95% CI 0.981–1.381; *p* = 0.081, statistical trend), whereas this association was not observed in patients with improved HF (i.e., prior HFrEF) (HR = 1.009; 95% CI 0.615–1.656; *p* = 0.971) (Supplemental Fig. 4). Finally, LVEF and NT-proBNP levels did not differ in patients with and without CKD in the presence of stable, deteriorated, or improved HF (Supplemental Fig. 5).

## Discussion

The present study investigates the prognostic impact of the severity and etiology of CKD in patients with HFmrEF using a large retrospective registry from 2016 to 2022. The data suggests that even milder stages of CKD (i.e., KDIGO stage 3a) were already associated with an increased risk of 30-month all-cause mortality compared to patients without CKD. This association was observed independently of the presence or absence of ischemic cardiomyopathy and confirmed after multivariable adjustment. In contrast, the long-term prognosis did not differ in patients with CKD and KDIGO stages 3b, 4, and 5. In contrast, patients with KDIGO stages 3b and 4 had the highest risk of HF-related rehospitalization at 30 months. Finally, the long-term prognosis was not affected by the etiology of CKD.

Related to ongoing demographic changes, the overall number of patients with HF suffering from cardiac and non-cardiac comorbidities is steadily increasing [6]. The prognostic value of CKD among patients with HFrEF, HFpEF, and HFmrEF was investigated within a large-scale sub-study of the “Swedish Heart Failure Registry.” In their study, the authors demonstrated an increased risk of all-cause mortality at 1 and at 5 years in patients with CKD, whereas this association was weaker in patients with HFpEF than in patients with HFrEF and HFmrEF [24]. However, in their study, the definition of CKD was based on a single creatinine and eGFR measurement leading to an overall increased prevalence of CKD (i.e., 45–56%). The overall higher rate of CKD compared to the contemporary study is in line with data from the BIOSTAT-CHF study [25]. This may be related to the fact that the definition of CKD required at least 2 eGFR measurements with a duration > 3 months for the present study. This may minimize the chance of including patients with acute kidney injury. In the present study, patients with CKD were accompanied by a risk of all-cause mortality of 34.0% at 1 year and 49.4% at 3 years, which was higher compared to previous studies in patients with HFmrEF (i.e., 23% at 1 year in the Swedish Heart Failure Registry) [24] and even higher compared to contemporary HFrEF registries (12.7% at 1 year in the Chang Gung Research Database) [26]. The mortality rates in the present study may be related to the all-comers setting of the present study and the absence of exclusion criteria, as well as the lower probability of including patients with acute kidney injury related to serial eGFR measurements. From this perspective, previous studies suggest an even higher risk of all-cause mortality in patients with CKD as compared to acute kidney injury [27].

When investigating the effect of different stages of CKD, the “Get With The Guidelines-Heart Failure” (GWTG-HF) suggested an increased risk of all-cause mortality in patients with HFmrEF and eGFR ≥ 60 mL/min/1.73 m^2^, whereas higher eGFR values were not associated with mortality; however, only minor part of the patients suffered from HFmrEF [28]. In line, data from the “RECOLFACA” registry revealed advanced stages of CKD (i.e., eGFR < 30 mL/min/1.73 m^2^) were associated with increased risk of all-cause mortality compared to patients with eGFR ≥ 60 mL/min/1.73 m^2^; however, the risk of all-cause mortality was not affected in patients with moderate CKD (i.e., GFR 30–59 mL/min/1.73 m^2^) [29]. The present study suggests that even milder stages of CKD were independently associated with an increased risk of 30-month all-cause mortality in consecutive patients with HFmrEF, which was confirmed using multivariable Cox regression analyses.

Of note, the risk of HF-related rehospitalization at 30 months was lower in patients with CKD and KDIGO stage 5 compared to patients with stages 3b and 4 within the present study. This may be associated with improved volume management in patients with renal replacement therapy, as well as the closer clinical follow-up in patients with end-stage renal disease. The “Continuous Ultrafiltration for Congestive Heart Failure” trial investigated the prognostic impact of ultrafiltration as compared to diuretic treatment in 56 patients with congestive HF. Although the risk of all-cause mortality at 1 year did not significantly differ in both groups, patients undergoing ultrafiltration had improved freedom from rehospitalization for HF and lower NT-proBNP levels compared to patients treated with diuretics [30]. In line with this, the “Aquapheresis versus Intravenous Diuretics and Hospitalization for Heart Failure” (AVOID-HF) trial demonstrated a longer time until the first HF-related rehospitalization at 90 days in patients undergoing ultrafiltration as compared to diuretic treatment only in 224 patients hospitalized for HF [31]. This may be in line with findings from the present study, suggesting improved freedom from HF-related rehospitalization in patients with CKD and KDIGO stage 5, whereas all patients required renal replacement therapy which may be in line with improved volume management as compared to diuretic treatment only. Finally, only a minor part of the study population in the present study was treated with sodium-glucose cotransporter 2 inhibitors (SGLT2i), which were shown to decrease the risk of cardiovascular mortality and HF-related rehospitalization in HFmrEF [32]. The low prescription rates of SGLT2i may be related to their recent upgraded level of evidence in 2023 and are supported by their low use in contemporary HFmrEF studies [11]. From this perspective, the “EMPEROR-Preserved” demonstrated the superiority of treatment with SGLT2i versus placebo, irrespective of the use and daily dose of concomitant diuretic treatment [33]. Furthermore, treatment with SGLT2i was associated with improved outcomes independent of CKD and KDIGO categories [34]. Further real-life studies are however needed to investigate the effect of CKD in HFmrEF following their inclusion into daily clinical practice.

## Study limitations

The study has several limitations. For the present study, at least two eGFR measurements separated by at least 3 months were required for the diagnosis of CKD in accordance with current international guidelines. Defining CKD based on the assessment of at least two eGFR measurements may, however, result in delayed or missed identification of patients with CKD in the absence of regular testing episodes within the present registry related to the consecutive inclusion of patients with HFmrEF. Furthermore, the inclusion of two eGFR values may lead to a survivor bias for fulfilling the criteria of eGFR according to the present guidelines [19]. In addition, patients with recurrent episodes of acute kidney injury may be misdiagnosed into the group of CKD. To decrease the chance of misclassification, eGFR values were considered following recompensation in patients with acute decompensated HF and multiple eGFR measurements during index hospitalization (> 95% patients with multiple eGFR measurements during index hospitalization within the present study). The presence of albuminuria was not assessed for the present study, although albuminuria was demonstrated to increase the risk of all-cause mortality among patients with CKD; therefore, the prevalence and prognosis of patients with early stages of CKD were beyond the scope of the present study [9]. HF-related and cardiac rehospitalization was assessed at our institution only. Finally, causes of death beyond during index hospitalization were not available for the present study.

## Conclusions

CKD represents one of the most common non-cardiovascular risk factors being present in 31% of patients with HFmrEF. Even milder stages of CKD were already independently associated with impaired long-term prognosis.

## Supplementary Information

Below is the link to the electronic supplementary material.

Supplementary file1 Supplemental Figure 1: Study flow chart (PPTX 40 KB)

Supplementary file2 Supplemental Figure 2: Kaplan-Meier analyses demonstrating the prognostic impact of different KDGIO stages on the primary endpoint all-cause mortality at 30 months (left panel), as well as on the risk of HF-related rehospitalization (right panel) stratified by patients with ischemic and non-ischemic cardiomyopathy (PPTX 159 KB)

Supplementary file3 Supplemental Figure 3: Changes in LVEF (left panel) and NT-pro BNP levels (right panel) among patients with and without CKD during 30 months stratified by patients with ischemic and non-ischemic cardiomyopathy (PPTX 396 KB)

Supplementary file4 Supplemental Figure 4: Kaplan-Meier analyses demonstrating the prognostic impact of different KDGIO stages on the primary endpoint all-cause mortality at 30 months (left panel), as well as on the risk of HF-related rehospitalization (right panel) stratified by patients with deteriorated, stable and improved LVEF (PPTX 197 KB)

Supplementary file5 Supplemental Figure 5: Changes in LVEF (left panel) and NT-pro BNP levels (right panel) among patients with and without CKD during 30 months stratified by patients with deteriorated, stable and improved LVEF (PPTX 567 KB)

Supplementary file6 Supplemental Table 1: Correlations of eGFR with clinical and laboratory data within the entire study cohort (DOCX 15 KB)

## References

[1] Bardy GH, Lee KL, Mark DB, Poole JE, Packer DL, Boineau R, Domanski M, Troutman C, Anderson J, Johnson G, McNulty SE, Clapp-Channing N, Davidson-Ray LD, Fraulo ES, Fishbein DP, Luceri RM, Ip JH (2005) Amiodarone or an implantable cardioverter-defibrillator for congestive heart failure. N Engl J Med 352(3):225–237 15659722 10.1056/NEJMoa043399

[2] Anker SD, Butler J, Filippatos G, Ferreira JP, Bocchi E, Böhm M, Brunner-La Rocca HP, Choi DJ, Chopra V, Chuquiure-Valenzuela E, Giannetti N, Gomez-Mesa JE, Janssens S, Januzzi JL, Gonzalez-Juanatey JR, Merkely B, Nicholls SJ, Perrone SV, Piña IL, Ponikowski P, Senni M, Sim D, Spinar J, Squire I, Taddei S, Tsutsui H, Verma S, Vinereanu D, Zhang J, Carson P, Lam CSP, Marx N, Zeller C, Sattar N, Jamal W, Schnaidt S, Schnee JM, Brueckmann M, Pocock SJ, Zannad F, Packer M (2021) Empagliflozin in heart failure with a preserved ejection fraction. N Engl J Med 385(16):1451–1461 34449189 10.1056/NEJMoa2107038

[3] Køber L, Torp-Pedersen C, Carlsen JE, Bagger H, Eliasen P, Lyngborg K, Videbaek J, Cole DS, Auclert L, Pauly NC (1995) A clinical trial of the angiotensin-converting-enzyme inhibitor trandolapril in patients with left ventricular dysfunction after myocardial infarction. Trandolapril Cardiac Evaluation (TRACE) Study Group. N Engl J Med 333(25):1670–6 7477219 10.1056/NEJM199512213332503

[4] McMurray JJ, Packer M, Desai AS, Gong J, Lefkowitz MP, Rizkala AR, Rouleau JL, Shi VC, Solomon SD, Swedberg K, Zile MR (2014) Angiotensin-neprilysin inhibition versus enalapril in heart failure. N Engl J Med 371(11):993–1004 25176015 10.1056/NEJMoa1409077

[5] Bollano E, Redfors B, Rawshani A, Venetsanos D, Völz S, Angerås O, Ljungman C, Alfredsson J, Jernberg T, Råmunddal T, Petursson P, Smith JG, Braun O, Hagström H, Fröbert O, Erlinge D, Omerovic E (2022) Temporal trends in characteristics and outcome of heart failure patients with and without significant coronary artery disease. ESC Heart Fail 9(3):1812–1822 35261201 10.1002/ehf2.13875 PMC9065869

[6] Chioncel O, Benson L, Crespo-Leiro MG, Anker SD, Coats AJS, Filippatos G, McDonagh T, Margineanu C, Mebazaa A, Metra M, Piepoli MF, Adamo M, Rosano GMC, Ruschitzka F, Savarese G, Seferovic P, Volterrani M, Ferrari R, Maggioni AP, Lund LH (2023) Comprehensive characterization of non-cardiac comorbidities in acute heart failure- an analysis of ESC-HFA EORP heart failure long-term registry. Eur J Prev Cardiol 30(13):1346–1358 37172316 10.1093/eurjpc/zwad151

[7] Tedeschi A, Agostoni P, Pezzuto B, Corra U, Scrutinio D, La Gioia R, Raimondo R, Passantino A, Piepoli MF (2020) Role of comorbidities in heart failure prognosis part 2: chronic kidney disease, elevated serum uric acid. Eur J Prev Cardiol 27(2_suppl):35–45 33238740 10.1177/2047487320957793 PMC7691631

[8] Weidner K, Behnes M, Schupp T, Rusnak J, Reiser L, Taton G, Reichelt T, Ellguth D, Engelke N, Bollow A, El-Battrawy I, Ansari U, Hoppner J, Nienaber CA, Mashayekhi K, Weiß C, Akin M, Borggrefe M, Akin I (2019) Prognostic impact of chronic kidney disease and renal replacement therapy in ventricular tachyarrhythmias and aborted cardiac arrest. Clin Res Cardiol 108(6):669–682 30578436 10.1007/s00392-018-1396-y

[9] Matsushita K, van der Velde M, Astor BC, Woodward M, Levey AS, de Jong PE, Coresh J, Gansevoort RT (2010) Association of estimated glomerular filtration rate and albuminuria with all-cause and cardiovascular mortality in general population cohorts: a collaborative meta-analysis. Lancet 375(9731):2073–2081 20483451 10.1016/S0140-6736(10)60674-5 PMC3993088

[10] McDonagh TA, Metra M, Adamo M, Gardner RS, Baumbach A, Böhm M, Burri H, Butler J, Čelutkienė J, Chioncel O, Cleland JGF, Coats AJS, Crespo-Leiro MG, Farmakis D, Gilard M, Heymans S, Hoes AW, Jaarsma T, Jankowska EA, Lainscak M, Lam CSP, Lyon AR, McMurray JJV, Mebazaa A, Mindham R, Muneretto C, Francesco Piepoli M, Price S, Rosano GMC, Ruschitzka F, Kathrine Skibelund A, Group ESD (2021) ESC guidelines for the diagnosis and treatment of acute and chronic heart failure: developed by the task force for the diagnosis and treatment of acute and chronic heart failure of the European Society of Cardiology (ESC) with the special contribution of the Heart Failure Association (HFA) of the ESC. Eur Heart J 42(36):3599–726 34649282 10.1093/eurheartj/ehab670

[11] McDonagh TA, Metra M, Adamo M, Gardner RS, Baumbach A, Böhm M, Burri H, Butler J, Čelutkienė J, Chioncel O, Cleland JGF, Crespo-Leiro MG, Farmakis D, Gilard M, Heymans S, Hoes AW, Jaarsma T, Jankowska EA, Lainscak M, Lam CSP, Lyon AR, McMurray JJV, Mebazaa A, Mindham R, Muneretto C, Francesco Piepoli M, Price S, Rosano GMC, Ruschitzka F, Skibelund AK, Group ESD (2023) 2023 Focused update of the 2021 ESC guidelines for the diagnosis and treatment of acute and chronic heart failure: developed by the task force for the diagnosis and treatment of acute and chronic heart failure of the European Society of Cardiology (ESC) with the special contribution of the Heart Failure Association (HFA) of the ESC. Eur Heart J 44(37):3627–3639 34649282 10.1093/eurheartj/ehab670

[12] Chioncel O, Lainscak M, Seferovic PM, Anker SD, Crespo-Leiro MG, Harjola VP, Parissis J, Laroche C, Piepoli MF, Fonseca C, Mebazaa A, Lund L, Ambrosio GA, Coats AJ, Ferrari R, Ruschitzka F, Maggioni AP, Filippatos G (2017) Epidemiology and one-year outcomes in patients with chronic heart failure and preserved, mid-range and reduced ejection fraction: an analysis of the ESC Heart Failure Long-Term Registry. Eur J Heart Fail 19(12):1574–1585 28386917 10.1002/ejhf.813

[13] Savarese G, Stolfo D, Sinagra G, Lund LH (2022) Heart failure with mid-range or mildly reduced ejection fraction. Nat Rev Cardiol 19(2):100–116 34489589 10.1038/s41569-021-00605-5 PMC8420965

[14] Chen S, Huang Z, Liang Y, Zhao X, Aobuliksimu X, Wang B, He Y, Kang Y, Huang H, Li Q, Yao Y, Lu X, Qian X, Xie X, Liu J, Liu Y (2022) Five-year mortality of heart failure with preserved, mildly reduced, and reduced ejection fraction in a 4880 Chinese cohort. ESC Heart Fail 9(4):2336–2347 35437939 10.1002/ehf2.13921 PMC9288761

[15] Escobar C, Palacios B, Varela L, Gutiérrez M, Duong M, Chen H, Justo N, Cid-Ruzafa J, Hernández I, Hunt PR, Delgado JF (2022) Prevalence, characteristics, management and outcomes of patients with heart failure with preserved, mildly reduced, and reduced ejection fraction in Spain. J Clin Med 11(17):5199 36079133 10.3390/jcm11175199 PMC9456780

[16] Schmitt A, Schupp T, Reinhardt M, Abel N, Lau F, Forner J, Ayoub M, Mashayekhi K, Weiß C, Akin I, Behnes M (2023) Prognostic impact of acute decompensated heart failure in patients with heart failure and mildly reduced ejection fraction. Eur Heart J Acute Cardiovasc 13(2):225–241 10.1093/ehjacc/zuad139 37950915

[17] Popescu BA, Andrade MJ, Badano LP, Fox KF, Flachskampf FA, Lancellotti P, Varga A, Sicari R, Evangelista A, Nihoyannopoulos P, Zamorano JL, Derumeaux G, Kasprzak JD, Roelandt JRTC, on behalf of the European Association of Echocardiography DR (2009) European Association of Echocardiography recommendations for training, competence, and quality improvement in echocardiography. European Journal of Echocardiography. 10(8):893–905 19889658 10.1093/ejechocard/jep151

[18] Lancellotti P, Tribouilloy C, Hagendorff A, Popescu BA, Edvardsen T, Pierard LA, Badano L, Zamorano JL (2013) Recommendations for the echocardiographic assessment of native valvular regurgitation: an executive summary from the European Association of Cardiovascular Imaging. Eur Heart J Cardiovasc Imaging 14(7):611–644 23733442 10.1093/ehjci/jet105

[19] Carrero JJ, Fu EL, Vestergaard SV, Jensen SK, Gasparini A, Mahalingasivam V, Bell S, Birn H, Heide-Jørgensen U, Clase CM, Cleary F, Coresh J, Dekker FW, Gansevoort RT, Hemmelgarn BR, Jager KJ, Jafar TH, Kovesdy CP, Sood MM, Stengel B, Christiansen CF, Iwagami M, Nitsch D (2023) Defining measures of kidney function in observational studies using routine health care data: methodological and reporting considerations. Kidney Int 103(1):53–69 36280224 10.1016/j.kint.2022.09.020

[20] Group KDIGOC-MW (2009) KDIGO clinical practice guideline for the diagnosis, evaluation, prevention, and treatment of chronic kidney disease-mineral and bone disorder (CKD-MBD). Kidney Int Suppl 76(113):S1-130 10.1038/ki.2009.188 19644521

[21] Andrassy KM (2013) Comments on ‘KDIGO 2012 clinical practice guideline for the evaluation and management of chronic kidney disease.’ Kidney Int 84(3):622–623 23989362 10.1038/ki.2013.243

[22] Levey AS, Stevens LA (2010) Estimating GFR using the CKD epidemiology collaboration (CKD-EPI) creatinine equation: more accurate GFR estimates, lower CKD prevalence estimates, and better risk predictions. Am J Kidney Dis 55(4):622–627 20338463 10.1053/j.ajkd.2010.02.337 PMC2846308

[23] Levey AS, Stevens LA, Schmid CH, Zhang YL, Castro AF 3rd, Feldman HI, Kusek JW, Eggers P, Van Lente F, Greene T, Coresh J (2009) A new equation to estimate glomerular filtration rate. Ann Intern Med 150(9):604–612 19414839 10.7326/0003-4819-150-9-200905050-00006 PMC2763564

[24] Löfman I, Szummer K, Dahlström U, Jernberg T, Lund LH (2017) Associations with and prognostic impact of chronic kidney disease in heart failure with preserved, mid-range, and reduced ejection fraction. Eur J Heart Fail 19(12):1606–1614 28371075 10.1002/ejhf.821

[25] Streng KW, Nauta JF, Hillege HL, Anker SD, Cleland JG, Dickstein K, Filippatos G, Lang CC, Metra M, Ng LL, Ponikowski P, Samani NJ, van Veldhuisen DJ, Zwinderman AH, Zannad F, Damman K, van der Meer P, Voors AA (2018) Non-cardiac comorbidities in heart failure with reduced, mid-range and preserved ejection fraction. Int J Cardiol 271:132–139 30482453 10.1016/j.ijcard.2018.04.001

[26] Lee WC, Liao TW, Chen TY, Fang HY, Fang YN, Chen HC, Lin YS, Chang SH, Chen MC (2023) Sacubitril/valsartan improves all-cause mortality in heart failure patients with reduced ejection fraction and chronic kidney disease. Cardiovasc Drugs Ther 10.1007/s10557-022-07421-0 PMC11101538 36609948

[27] Yandrapalli S, Christy J, Malik A, Wats K, Harikrishnan P, Aronow W, Frishman W (2022) Impact of acute and chronic kidney disease on heart failure hospitalizations after acute myocardial infarction. Am J Cardiol 165:1–11 34893301 10.1016/j.amjcard.2021.10.041

[28] Patel RB, Fonarow GC, Greene SJ, Zhang S, Alhanti B, DeVore AD, Butler J, Heidenreich PA, Huang JC, Kittleson MM, Joynt Maddox KE, McDermott JJ, Owens AT, Peterson PN, Solomon SD, Vardeny O, Yancy CW, Vaduganathan M (2021) Kidney function and outcomes in patients hospitalized with heart failure. J Am Coll Cardiol 78(4):330–343 33989713 10.1016/j.jacc.2021.05.002 PMC8994871

[29] López-Ponce de León JD, Gómez-Mesa JE, Saldarriaga C, Echeverría LE, Posada-Bastidas A, García JC, Ochoa-Morón AD, Rolong B, Manzur-Jatin F, Mosquera-Jiménez JI, Pacheco-Jiménez OA, Rodríguez-Cerón ÁH, Rodríguez-Gómez P, Rivera-Toquica F, Rivera-Toquica A (2023) Prevalence, clinical characteristics and prognostic impact of kidney disease on heart failure patients: an observational study of the Colombian Heart Failure Registry (RECOLFACA). Cardiorenal Med 1:292–300 10.1159/000530852 37231884

[30] Marenzi G, Muratori M, Cosentino ER, Rinaldi ER, Donghi V, Milazzo V, Ferramosca E, Borghi C, Santoro A, Agostoni P (2014) Continuous ultrafiltration for congestive heart failure: the CUORE trial. J Card Fail 20(1):9–17 24269855 10.1016/j.cardfail.2013.11.004

[31] Costanzo MR, Negoianu D, Jaski BE, Bart BA, Heywood JT, Anand IS, Smelser JM, Kaneshige AM, Chomsky DB, Adler ED, Haas GJ, Watts JA, Nabut JL, Schollmeyer MP, Fonarow GC (2016) Aquapheresis Versus Intravenous Diuretics and Hospitalizations for Heart Failure. JACC Heart Fail 4(2):95–105 26519995 10.1016/j.jchf.2015.08.005

[32] Anker SD, Butler J, Filippatos G, Ferreira JP, Bocchi E, Böhm M, Brunner–La Rocca H-P, Choi D-J, Chopra V, Chuquiure-Valenzuela E, Giannetti N, Gomez-Mesa JE, Janssens S, Januzzi JL, Gonzalez-Juanatey JR, Merkely B, Nicholls SJ, Perrone SV, Piña IL, Ponikowski P, Senni M, Sim D, Spinar J, Squire I, Taddei S, Tsutsui H, Verma S, Vinereanu D, Zhang J, Carson P, Lam CSP, Marx N, Zeller C, Sattar N, Jamal W, Schnaidt S, Schnee JM, Brueckmann M, Pocock SJ, Zannad F, Packer M (2021) Empagliflozin in heart failure with a preserved ejection fraction. N Engl J Med 385(16):1451–61 34449189 10.1056/NEJMoa2107038

[33] Butler J, Usman MS, Filippatos G, Ferreira JP, Böhm M, Brueckmann M, Januzzi JL, Kaul S, Piña IL, Ponikowski P, Senni M, Sumin M, Verma S, Zaremba-Pechmann L, Pocock SJ, Packer M, Anker S (2023) Safety and efficacy of empagliflozin and diuretic use in patients with heart failure and preserved ejection fraction: a post hoc analysis of the EMPEROR-preserved trial. JAMA Cardiol 8(7):640–649 37223933 10.1001/jamacardio.2023.1090 PMC10209829

[34] Butler J, Packer M, Siddiqi TJ, Böhm M, Brueckmann M, Januzzi JL, Verma S, Gergei I, Iwata T, Wanner C, Ferreira JP, Pocock SJ, Filippatos G, Anker SD, Zannad F (2023) Efficacy of empagliflozin in patients with heart failure across kidney risk categories. J Am Coll Cardiol 81(19):1902–1914 37164523 10.1016/j.jacc.2023.03.390

