# Supplementary material for: Effect of severity and etiology of chronic kidney disease in patients with heart failure with mildly reduced ejection fraction
Source: Clin Res Cardiol. 2024 May 6;113(11):1565–75. doi: 10.1007/s00392-024-02453-y (PMC11493827; doi:10.1007/s00392-024-02453-y)
Supplement: Supplementary file 1 — Supplementary file1 Supplemental Figure 1: Study flow chart (PPTX 40 KB) [file 392_2024_2453_MOESM1_ESM.pptx]

## Slide 1
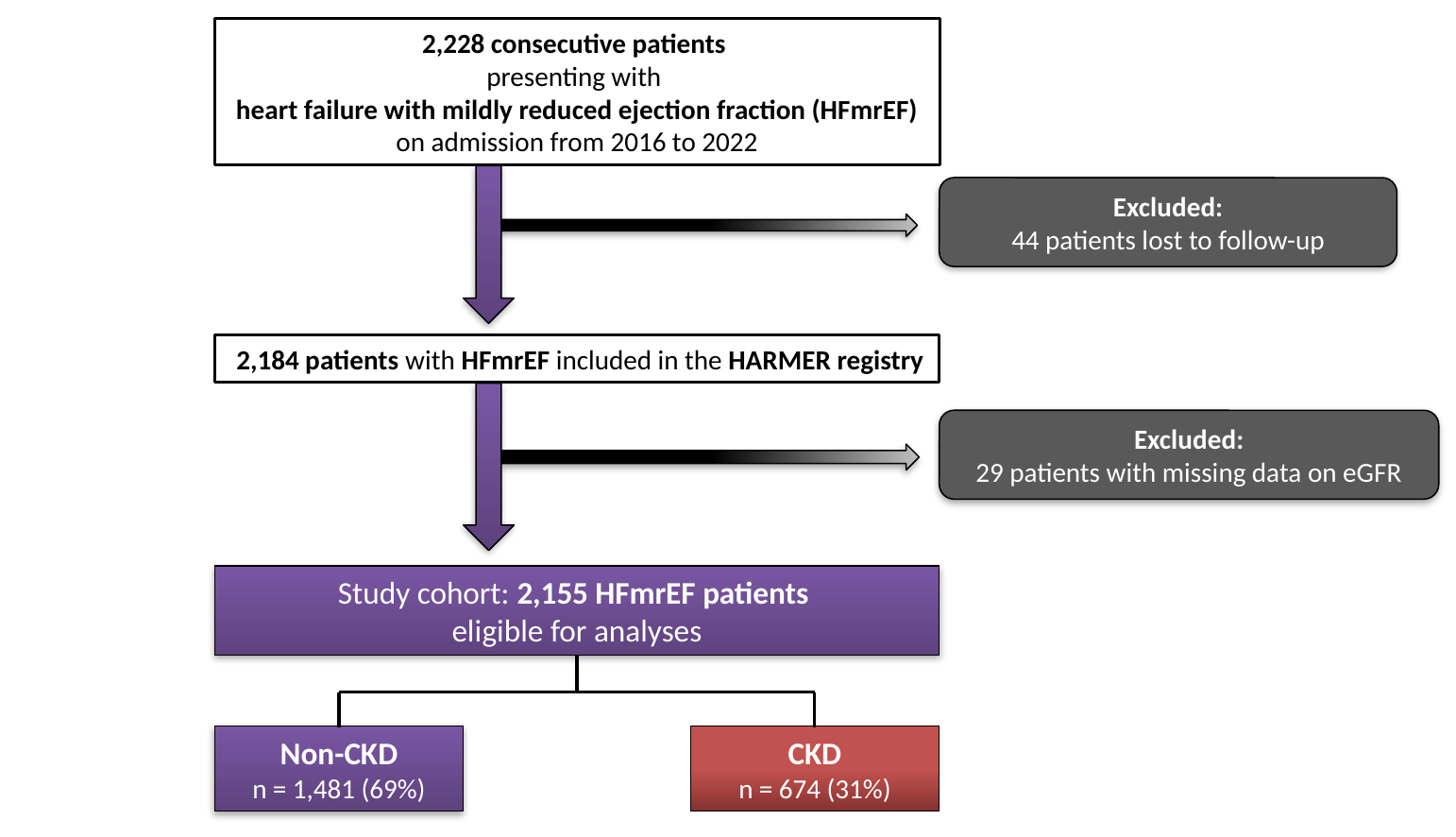

2,228 consecutive patients
presenting with
heart failure with mildly reduced ejection fraction (HFmrEF)
on admission from 2016 to 2022
Excluded:
44 patients lost to follow-up
 2,184 patients with HFmrEF included in the HARMER registry
Excluded:
29 patients with missing data on eGFR
Study cohort: 2,155 HFmrEF patients
eligible for analyses
Non-CKD
n = 1,481 (69%)
CKD
n = 674 (31%)
