# Supplementary material for: Effect of severity and etiology of chronic kidney disease in patients with heart failure with mildly reduced ejection fraction
Source: Clin Res Cardiol. 2024 May 6;113(11):1565–75. doi: 10.1007/s00392-024-02453-y (PMC11493827; doi:10.1007/s00392-024-02453-y)
Supplement: Supplementary file 2 — Supplementary file2 Supplemental Figure 2: Kaplan-Meier analyses demonstrating the prognostic impact of different KDGIO stages on the primary endpoint all-cause mortality at 30 months (left panel), as well as on the risk of HF-related rehospitalization (right panel) stratified by patients with ischemic and non-ischemic cardiomyopathy (PPTX 159 KB) [file 392_2024_2453_MOESM2_ESM.pptx]

## Slide 1
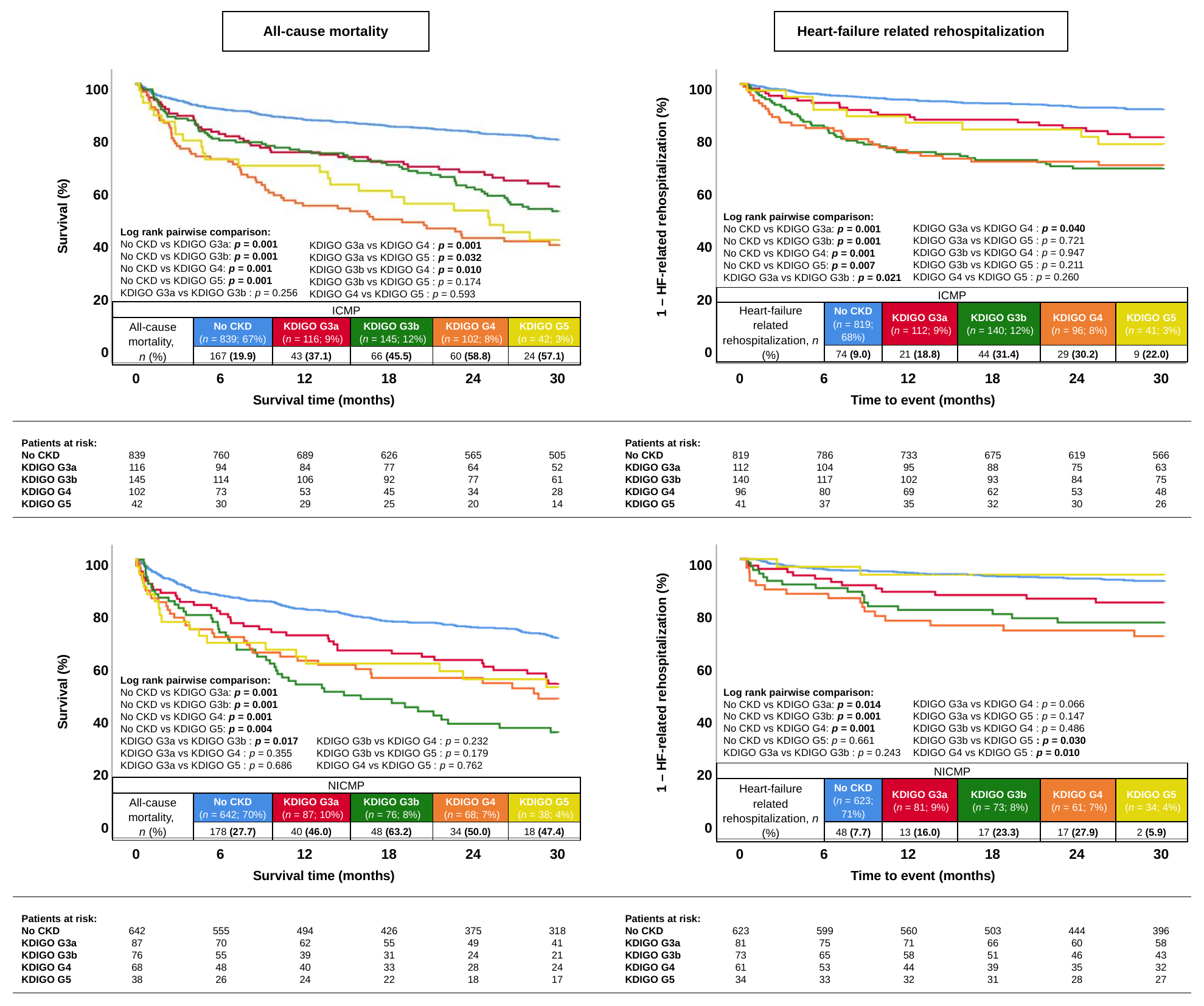

| All-cause mortality |
| --- |
| Heart-failure related rehospitalization |
| --- |
100
80
60
40
20
0
100
80
60
40
20
0
1 – HF-related rehospitalization (%)
Survival (%)
Log rank pairwise comparison:
No CKD vs KDIGO G3a: p = 0.001
No CKD vs KDIGO G3b: p = 0.001
No CKD vs KDIGO G4: p = 0.001
No CKD vs KDIGO G5: p = 0.007
KDIGO G3a vs KDIGO G3b : p = 0.021
KDIGO G3a vs KDIGO G4 : p = 0.040
KDIGO G3a vs KDIGO G5 : p = 0.721
KDIGO G3b vs KDIGO G4 : p = 0.947
KDIGO G3b vs KDIGO G5 : p = 0.211
KDIGO G4 vs KDIGO G5 : p = 0.260
Log rank pairwise comparison:
No CKD vs KDIGO G3a: p = 0.001
No CKD vs KDIGO G3b: p = 0.001
No CKD vs KDIGO G4: p = 0.001
No CKD vs KDIGO G5: p = 0.001
KDIGO G3a vs KDIGO G3b : p = 0.256
KDIGO G3a vs KDIGO G4 : p = 0.001
KDIGO G3a vs KDIGO G5 : p = 0.032
KDIGO G3b vs KDIGO G4 : p = 0.010
KDIGO G3b vs KDIGO G5 : p = 0.174
KDIGO G4 vs KDIGO G5 : p = 0.593
| ICMP | | | | | |
| --- | --- | --- | --- | --- | --- |
| Heart-failure related rehospitalization, n (%) | No CKD (n = 819; 68%) | KDIGO G3a (n = 112; 9%) | KDIGO G3b (n = 140; 12%) | KDIGO G4 (n = 96; 8%) | KDIGO G5 (n = 41; 3%) |
| | 74 (9.0) | 21 (18.8) | 44 (31.4) | 29 (30.2) | 9 (22.0) |
| ICMP | | | | | |
| --- | --- | --- | --- | --- | --- |
| All-cause mortality, n (%) | No CKD (n = 839; 67%) | KDIGO G3a (n = 116; 9%) | KDIGO G3b (n = 145; 12%) | KDIGO G4 (n = 102; 8%) | KDIGO G5 (n = 42; 3%) |
| | 167 (19.9) | 43 (37.1) | 66 (45.5) | 60 (58.8) | 24 (57.1) |
0
6
12
18
24
30
0
6
12
18
24
30
Survival time (months)
Time to event (months)
Patients at risk:
No CKD
KDIGO G3a
KDIGO G3b
KDIGO G4
KDIGO G5
Patients at risk:
No CKD
KDIGO G3a
KDIGO G3b
KDIGO G4
KDIGO G5
689
84
106
53
29
626
77
92
45
25
565
64
77
34
20
505
52
61
28
14
733
95
102
69
35
675
88
93
62
32
619
75
84
53
30
566
63
75
48
26
839
116
145
102
42
760
94
114
73
30
819
112
140
96
41
786
104
117
80
37
100
80
60
40
20
0
100
80
60
40
20
0
Log rank pairwise comparison:
No CKD vs KDIGO G3a: p = 0.001
No CKD vs KDIGO G3b: p = 0.001
No CKD vs KDIGO G4: p = 0.001
No CKD vs KDIGO G5: p = 0.004
KDIGO G3a vs KDIGO G3b : p = 0.017
KDIGO G3a vs KDIGO G4 : p = 0.355
KDIGO G3a vs KDIGO G5 : p = 0.686
1 – HF-related rehospitalization (%)
Survival (%)
Log rank pairwise comparison:
No CKD vs KDIGO G3a: p = 0.014
No CKD vs KDIGO G3b: p = 0.001
No CKD vs KDIGO G4: p = 0.001
No CKD vs KDIGO G5: p = 0.661
KDIGO G3a vs KDIGO G3b : p = 0.243
KDIGO G3a vs KDIGO G4 : p = 0.066
KDIGO G3a vs KDIGO G5 : p = 0.147
KDIGO G3b vs KDIGO G4 : p = 0.486
KDIGO G3b vs KDIGO G5 : p = 0.030
KDIGO G4 vs KDIGO G5 : p = 0.010
KDIGO G3b vs KDIGO G4 : p = 0.232
KDIGO G3b vs KDIGO G5 : p = 0.179
KDIGO G4 vs KDIGO G5 : p = 0.762
| NICMP | | | | | |
| --- | --- | --- | --- | --- | --- |
| Heart-failure related rehospitalization, n (%) | No CKD (n = 623; 71%) | KDIGO G3a (n = 81; 9%) | KDIGO G3b (n = 73; 8%) | KDIGO G4 (n = 61; 7%) | KDIGO G5 (n = 34; 4%) |
| | 48 (7.7) | 13 (16.0) | 17 (23.3) | 17 (27.9) | 2 (5.9) |
| NICMP | | | | | |
| --- | --- | --- | --- | --- | --- |
| All-cause mortality, n (%) | No CKD (n = 642; 70%) | KDIGO G3a (n = 87; 10%) | KDIGO G3b (n = 76; 8%) | KDIGO G4 (n = 68; 7%) | KDIGO G5 (n = 38; 4%) |
| | 178 (27.7) | 40 (46.0) | 48 (63.2) | 34 (50.0) | 18 (47.4) |
0
6
12
18
24
30
0
6
12
18
24
30
Survival time (months)
Time to event (months)
Patients at risk:
No CKD
KDIGO G3a
KDIGO G3b
KDIGO G4
KDIGO G5
Patients at risk:
No CKD
KDIGO G3a
KDIGO G3b
KDIGO G4
KDIGO G5
494
62
39
40
24
426
55
31
33
22
375
49
24
28
18
318
41
21
24
17
560
71
58
44
32
503
66
51
39
31
444
60
46
35
28
396
58
43
32
27
642
87
76
68
38
555
70
55
48
26
623
81
73
61
34
599
75
65
53
33
