# Supplementary figures and images for: Effect of severity and etiology of chronic kidney disease in patients with heart failure with mildly reduced ejection fraction
Source: Clin Res Cardiol. 2024 May 6;113(11):1565–75. doi: 10.1007/s00392-024-02453-y (PMC11493827; doi:10.1007/s00392-024-02453-y)

## Slide 1
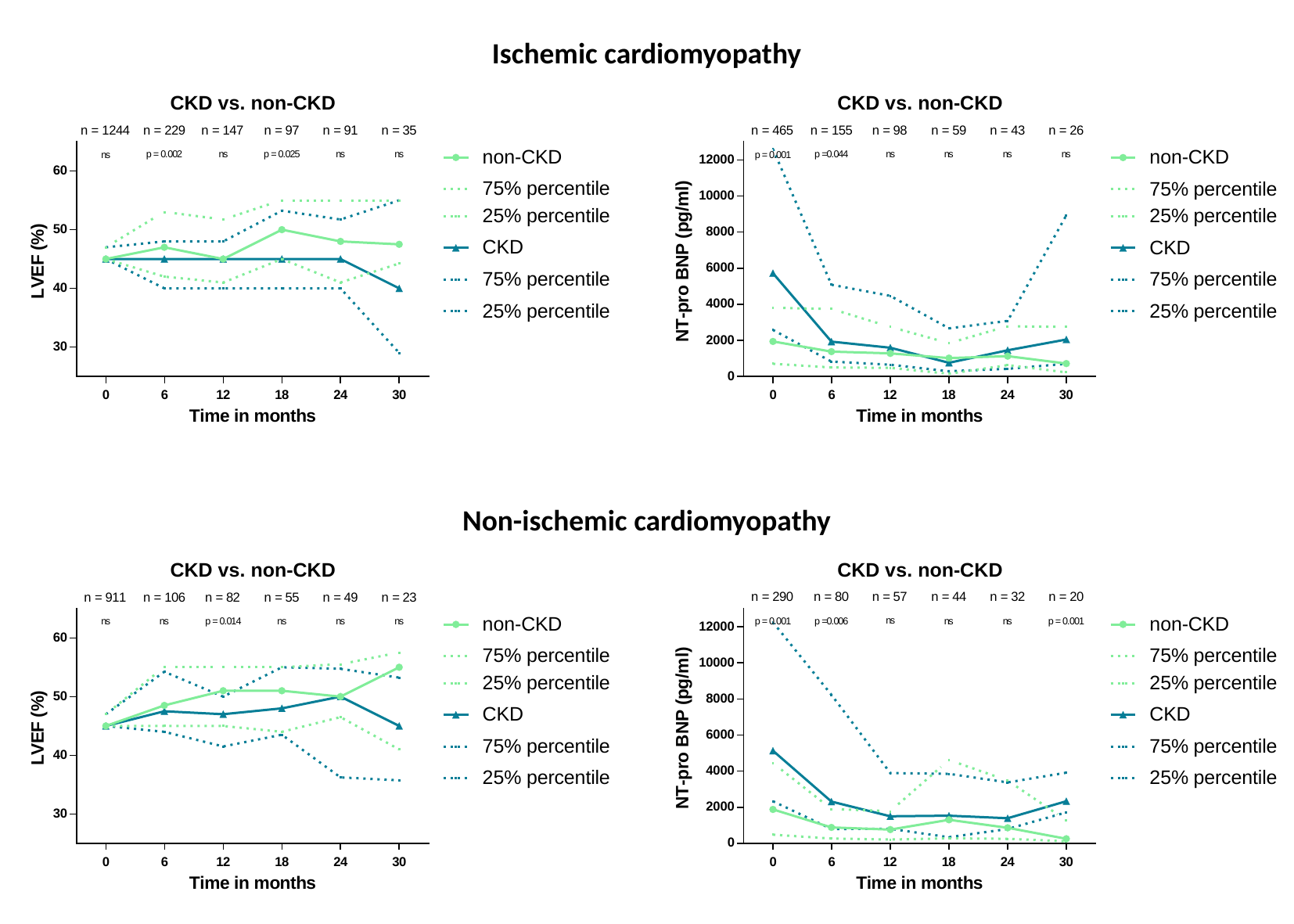

Ischemic cardiomyopathy
Non-ischemic cardiomyopathy

Supplement: Supplementary file 3 — Supplementary file3 Supplemental Figure 3: Changes in LVEF (left panel) and NT-pro BNP levels (right panel) among patients with and without CKD during 30 months stratified by patients with ischemic and non-ischemic cardiomyopathy (PPTX 396 KB) [file 392_2024_2453_MOESM3_ESM.pptx]

## Slide 1
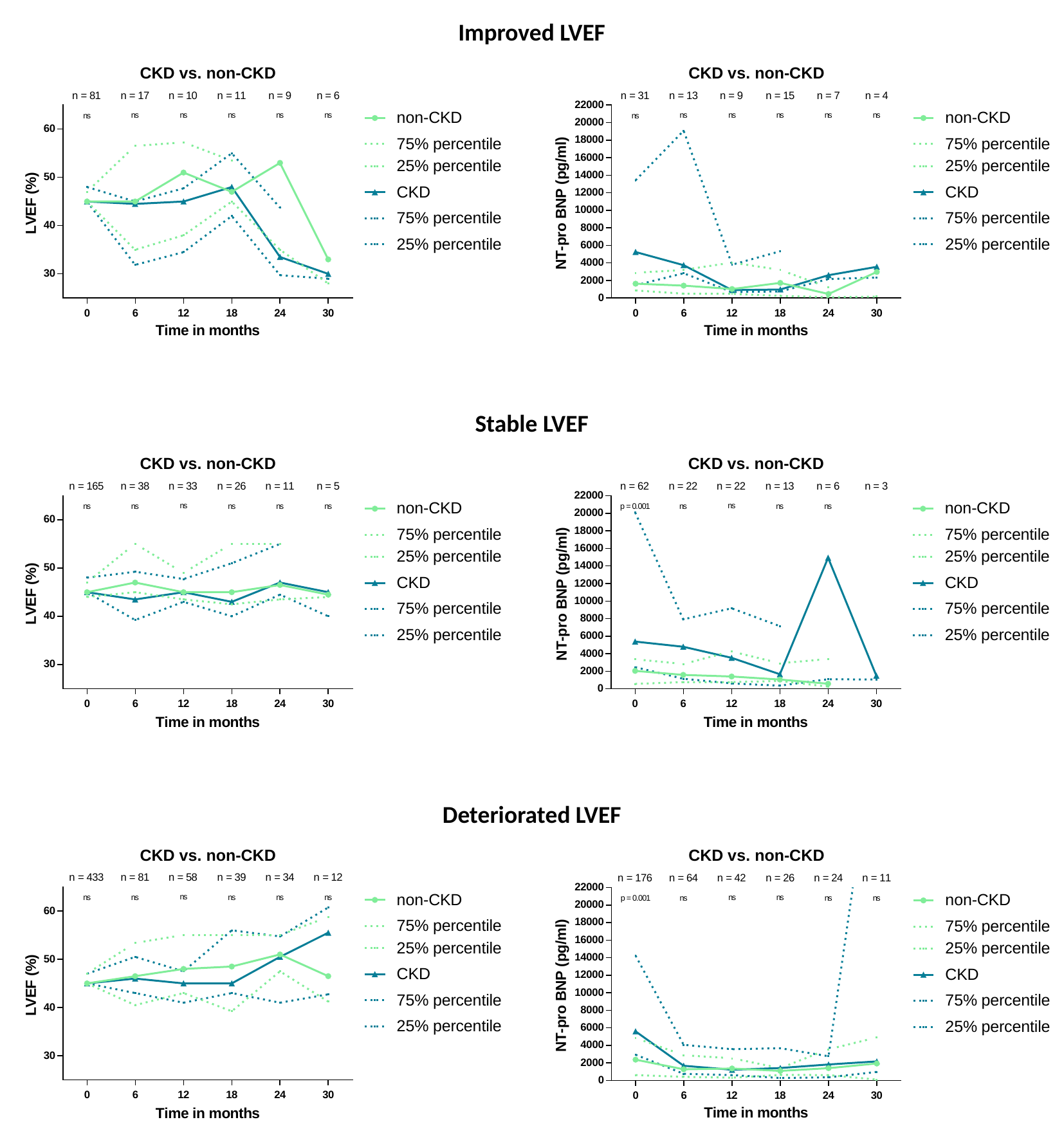

Improved LVEF
Stable LVEF
Deteriorated LVEF

Supplement: Supplementary file 5 — Supplementary file5 Supplemental Figure 5: Changes in LVEF (left panel) and NT-pro BNP levels (right panel) among patients with and without CKD during 30 months stratified by patients with deteriorated, stable and improved LVEF (PPTX 567 KB) [file 392_2024_2453_MOESM5_ESM.pptx]
