# Supplementary material for: Effect of severity and etiology of chronic kidney disease in patients with heart failure with mildly reduced ejection fraction
Source: Clin Res Cardiol. 2024 May 6;113(11):1565–75. doi: 10.1007/s00392-024-02453-y (PMC11493827; doi:10.1007/s00392-024-02453-y)
Supplement: Supplementary file 4 — Supplementary file4 Supplemental Figure 4: Kaplan-Meier analyses demonstrating the prognostic impact of different KDGIO stages on the primary endpoint all-cause mortality at 30 months (left panel), as well as on the risk of HF-related rehospitalization (right panel) stratified by patients with deteriorated, stable and improved LVEF (PPTX 197 KB) [file 392_2024_2453_MOESM4_ESM.pptx]

## Slide 1
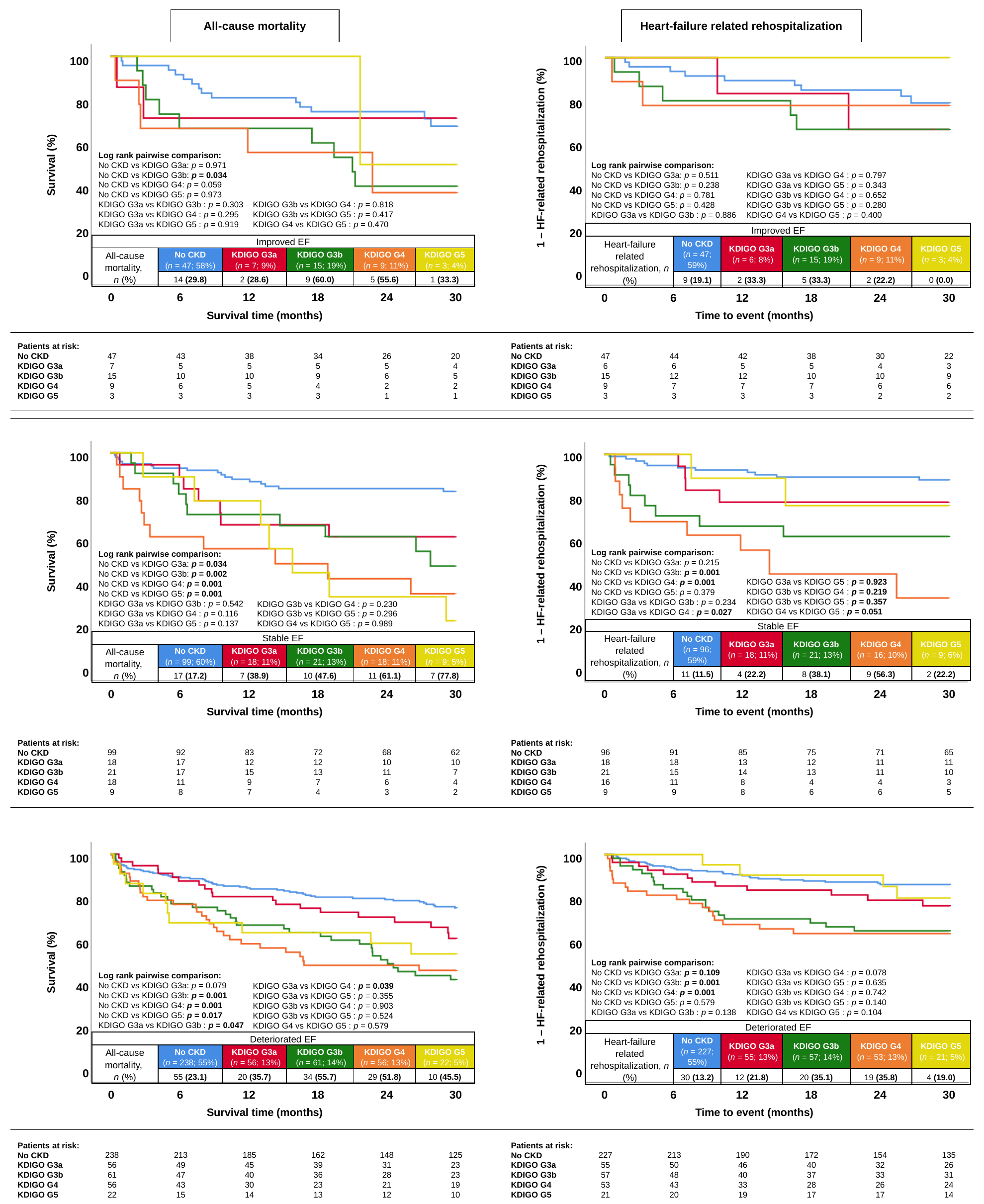

| All-cause mortality |
| --- |
| Heart-failure related rehospitalization |
| --- |
100
80
60
40
20
0
100
80
60
40
20
0
Log rank pairwise comparison:
No CKD vs KDIGO G3a: p = 0.971
No CKD vs KDIGO G3b: p = 0.034
No CKD vs KDIGO G4: p = 0.059
No CKD vs KDIGO G5: p = 0.973
KDIGO G3a vs KDIGO G3b : p = 0.303
KDIGO G3a vs KDIGO G4 : p = 0.295
KDIGO G3a vs KDIGO G5 : p = 0.919
1 – HF-related rehospitalization (%)
Survival (%)
Log rank pairwise comparison:
No CKD vs KDIGO G3a: p = 0.511
No CKD vs KDIGO G3b: p = 0.238
No CKD vs KDIGO G4: p = 0.781
No CKD vs KDIGO G5: p = 0.428
KDIGO G3a vs KDIGO G3b : p = 0.886
KDIGO G3a vs KDIGO G4 : p = 0.797
KDIGO G3a vs KDIGO G5 : p = 0.343
KDIGO G3b vs KDIGO G4 : p = 0.652
KDIGO G3b vs KDIGO G5 : p = 0.280
KDIGO G4 vs KDIGO G5 : p = 0.400
KDIGO G3b vs KDIGO G4 : p = 0.818
KDIGO G3b vs KDIGO G5 : p = 0.417
KDIGO G4 vs KDIGO G5 : p = 0.470
| Improved EF | | | | | |
| --- | --- | --- | --- | --- | --- |
| Heart-failure related rehospitalization, n (%) | No CKD (n = 47; 59%) | KDIGO G3a (n = 6; 8%) | KDIGO G3b (n = 15; 19%) | KDIGO G4 (n = 9; 11%) | KDIGO G5 (n = 3; 4%) |
| | 9 (19.1) | 2 (33.3) | 5 (33.3) | 2 (22.2) | 0 (0.0) |
| Improved EF | | | | | |
| --- | --- | --- | --- | --- | --- |
| All-cause mortality, n (%) | No CKD (n = 47; 58%) | KDIGO G3a (n = 7; 9%) | KDIGO G3b (n = 15; 19%) | KDIGO G4 (n = 9; 11%) | KDIGO G5 (n = 3; 4%) |
| | 14 (29.8) | 2 (28.6) | 9 (60.0) | 5 (55.6) | 1 (33.3) |
0
6
12
18
24
30
0
6
12
18
24
30
Survival time (months)
Time to event (months)
Patients at risk:
No CKD
KDIGO G3a
KDIGO G3b
KDIGO G4
KDIGO G5
Patients at risk:
No CKD
KDIGO G3a
KDIGO G3b
KDIGO G4
KDIGO G5
38
5
10
5
3
34
5
9
4
3
26
5
6
2
1
20
4
5
2
1
42
5
12
7
3
38
5
10
7
3
30
4
10
6
2
22
3
9
6
2
47
7
15
9
3
43
5
10
6
3
47
6
15
9
3
44
6
12
7
3
100
80
60
40
20
0
100
80
60
40
20
0
1 – HF-related rehospitalization (%)
Log rank pairwise comparison:
No CKD vs KDIGO G3a: p = 0.215
No CKD vs KDIGO G3b: p = 0.001
No CKD vs KDIGO G4: p = 0.001
No CKD vs KDIGO G5: p = 0.379
KDIGO G3a vs KDIGO G3b : p = 0.234
KDIGO G3a vs KDIGO G4 : p = 0.027
Log rank pairwise comparison:
No CKD vs KDIGO G3a: p = 0.034
No CKD vs KDIGO G3b: p = 0.002
No CKD vs KDIGO G4: p = 0.001
No CKD vs KDIGO G5: p = 0.001
KDIGO G3a vs KDIGO G3b : p = 0.542
KDIGO G3a vs KDIGO G4 : p = 0.116
KDIGO G3a vs KDIGO G5 : p = 0.137
Survival (%)
KDIGO G3a vs KDIGO G5 : p = 0.923
KDIGO G3b vs KDIGO G4 : p = 0.219
KDIGO G3b vs KDIGO G5 : p = 0.357
KDIGO G4 vs KDIGO G5 : p = 0.051
KDIGO G3b vs KDIGO G4 : p = 0.230
KDIGO G3b vs KDIGO G5 : p = 0.296
KDIGO G4 vs KDIGO G5 : p = 0.989
| Stable EF | | | | | |
| --- | --- | --- | --- | --- | --- |
| Heart-failure related rehospitalization, n (%) | No CKD (n = 96; 59%) | KDIGO G3a (n = 18; 11%) | KDIGO G3b (n = 21; 13%) | KDIGO G4 (n = 16; 10%) | KDIGO G5 (n = 9; 6%) |
| | 11 (11.5) | 4 (22.2) | 8 (38.1) | 9 (56.3) | 2 (22.2) |
| Stable EF | | | | | |
| --- | --- | --- | --- | --- | --- |
| All-cause mortality, n (%) | No CKD (n = 99; 60%) | KDIGO G3a (n = 18; 11%) | KDIGO G3b (n = 21; 13%) | KDIGO G4 (n = 18; 11%) | KDIGO G5 (n = 9; 5%) |
| | 17 (17.2) | 7 (38.9) | 10 (47.6) | 11 (61.1) | 7 (77.8) |
0
6
12
18
24
30
0
6
12
18
24
30
Survival time (months)
Time to event (months)
Patients at risk:
No CKD
KDIGO G3a
KDIGO G3b
KDIGO G4
KDIGO G5
Patients at risk:
No CKD
KDIGO G3a
KDIGO G3b
KDIGO G4
KDIGO G5
83
12
15
9
7
72
12
13
7
4
68
10
11
6
3
62
10
7
4
2
85
13
14
8
8
75
12
13
4
6
71
11
11
4
6
65
11
10
3
5
99
18
21
18
9
92
17
17
11
8
96
18
21
16
9
91
18
15
11
9
100
80
60
40
20
0
100
80
60
40
20
0
1 – HF-related rehospitalization (%)
Survival (%)
Log rank pairwise comparison:
No CKD vs KDIGO G3a: p = 0.109
No CKD vs KDIGO G3b: p = 0.001
No CKD vs KDIGO G4: p = 0.001
No CKD vs KDIGO G5: p = 0.579
KDIGO G3a vs KDIGO G3b : p = 0.138
KDIGO G3a vs KDIGO G4 : p = 0.078
KDIGO G3a vs KDIGO G5 : p = 0.635
KDIGO G3b vs KDIGO G4 : p = 0.742
KDIGO G3b vs KDIGO G5 : p = 0.140
KDIGO G4 vs KDIGO G5 : p = 0.104
Log rank pairwise comparison:
No CKD vs KDIGO G3a: p = 0.079
No CKD vs KDIGO G3b: p = 0.001
No CKD vs KDIGO G4: p = 0.001
No CKD vs KDIGO G5: p = 0.017
KDIGO G3a vs KDIGO G3b : p = 0.047
KDIGO G3a vs KDIGO G4 : p = 0.039
KDIGO G3a vs KDIGO G5 : p = 0.355
KDIGO G3b vs KDIGO G4 : p = 0.903
KDIGO G3b vs KDIGO G5 : p = 0.524
KDIGO G4 vs KDIGO G5 : p = 0.579
| Deteriorated EF | | | | | |
| --- | --- | --- | --- | --- | --- |
| Heart-failure related rehospitalization, n (%) | No CKD (n = 227; 55%) | KDIGO G3a (n = 55; 13%) | KDIGO G3b (n = 57; 14%) | KDIGO G4 (n = 53; 13%) | KDIGO G5 (n = 21; 5%) |
| | 30 (13.2) | 12 (21.8) | 20 (35.1) | 19 (35.8) | 4 (19.0) |
| Deteriorated EF | | | | | |
| --- | --- | --- | --- | --- | --- |
| All-cause mortality, n (%) | No CKD (n = 238; 55%) | KDIGO G3a (n = 56; 13%) | KDIGO G3b (n = 61; 14%) | KDIGO G4 (n = 56; 13%) | KDIGO G5 (n = 22; 5%) |
| | 55 (23.1) | 20 (35.7) | 34 (55.7) | 29 (51.8) | 10 (45.5) |
0
6
12
18
24
30
0
6
12
18
24
30
Survival time (months)
Time to event (months)
Patients at risk:
No CKD
KDIGO G3a
KDIGO G3b
KDIGO G4
KDIGO G5
Patients at risk:
No CKD
KDIGO G3a
KDIGO G3b
KDIGO G4
KDIGO G5
185
45
40
30
14
162
39
36
23
13
148
31
28
21
12
125
23
23
19
10
190
46
40
33
19
172
40
37
28
17
154
32
33
26
17
135
26
31
24
14
238
56
61
56
22
213
49
47
43
15
227
55
57
53
21
213
50
48
43
20
