# Supplementary material for: Effect of severity and etiology of chronic kidney disease in patients with heart failure with mildly reduced ejection fraction
Source: Clin Res Cardiol. 2024 May 6;113(11):1565–75. doi: 10.1007/s00392-024-02453-y (PMC11493827; doi:10.1007/s00392-024-02453-y)
Supplement: Supplementary file 6 — Supplementary file6 Supplemental Table 1: Correlations of eGFR with clinical and laboratory data within the entire study cohort (DOCX 15 KB) [file 392_2024_2453_MOESM6_ESM.docx]

| Supplemental Table 1. Correlations of eGFR with laboratory and clinical parameters. | | |
| --- | --- | --- |
|  | eGFR | |
|  | r | p value |
| Age | -0.361 | 0.001 |
| Body mass index (kg/m^2^) | 0.000 | 0.989 |
| Sodium (mmol/L) | 0.010 | 0.655 |
| Potassium (mmol/L) | -0.177 | 0.001 |
| Hemoglobin (g/dL) | 0.340 | 0.001 |
| WBC count (x 10^9^/L) | -0.018 | 0.412 |
| Platelet count (x 10^9^/L) | 0.105 | 0.001 |
| NT-pro BNP (pg/mL) | -0.141 | 0.001 |
| Cardiac troponin I (µg/L) | -0.003 | 0.891 |
| Total cholesterol (mg/dL) | 0.147 | 0.001 |
| LDL (mg/dL) | 0 0.195 | 0.001 |
| HDL (mg/dL) | 0.025 | 0.404 |
| HbA1c (%) | -0.194 | 0.001 |
| C-reactive protein (mg/L) | -0.144 | 0.001 |
| Procalcitonin (µg/L) | -0.287 | 0.001 |
| eGFR, estimated glomerular filtration rate; HbA1c, glycated haemoglobin; HDL, high-density lipoprotein; LDL, low-density lipoprotein; LVEDD, left ventricular end-diastolic diameter; NT-pro BNP, aminoterminal pro-B-type natriuretic peptide; WBC, white blood cell count. Level of significance p<0.05. Bold type indicates statistical significance. | | |

.
